# Trajectories, Turning Points, and Interruptions: How Social Inequality Shapes Women’s Later-Life Health

**DOI:** 10.1093/geroni/igab046.1958

**Published:** 2021-12-17

**Authors:** Jessica Kelley, Marissa Gilbert

**Affiliations:** 1 Case Western Reserve University, Celeveland, Ohio, United States; 2 Case Western Reserve University, Cleveland, Ohio, United States

## Abstract

We explore women’s health in midlife and later life at the nexus of structural sexism and the life course perspective, applying Dannefer’s (2018) concept of life course reflexivity, which emphasizes social dynamism with potential health-changing ‘input’ at all ages. We present three types of reflexive changes in the gendered life course that shape women’s health as they age: (1) trajectories of lifetime labor market disadvantage leading to limited health-protective resources in later life; (2) turning points in family structure and need, with draining caregiving demands; (3) interruptions in midlife, such as divorce, erasing the social and economic benefits of marriage. We provide support for critical arguments that theoretical work on the life course has too-often utilized the ‘privileged’ or the ‘male’ life course with insufficient attention to structural sexism as a fundamental cause of women’s health disparities in later life.

